# Pharmacophore Variants of the Macrocyclic Peptide Triazole Inactivator of HIV-1 Env

**DOI:** 10.21203/rs.3.rs-2814722/v1

**Published:** 2023-04-20

**Authors:** Monisha Gupta, Gabriela Canziani, Charles Ang, Mohammadjavad Mohammadi, Cameron F. Abrams, Derek Yang, Amos B. Smith, Irwin Chaiken

**Affiliations:** 1 Department of Biochemistry and Molecular Biology, College of Medicine, Drexel University, Philadelphia, Pennsylvania 19102, United States; 2 Department of Chemistry, College of Arts and Sciences, Drexel University, Philadelphia, Pennsylvania 19102, United States; 3 Department of Chemical & Biological Engineering, College of Engineering, Drexel University, Philadelphia, Pennsylvania 19102, United States; 4 Department of Chemistry, School of Arts and Sciences, University of Pennsylvania, Philadelphia, Pennsylvania 19104, United States

## Abstract

Previously we established a family of macrocyclic peptide triazoles (cPTs) that inactivate the Env protein complex of HIV-1, and identified the pharmacophore that engages Env’s receptor binding pocket. Here, we examined the hypothesis that the side chains of both components of the triazole Pro - Trp segment of cPT pharmacophore work in tandem to make intimate contacts with two proximal subsites of the overall CD4 binding site of gp120 to stabilize binding and function. Variations of the triazole Pro R group, which previously had been significantly optimized, led to identification of a variant MG-II-20 that contains a pyrazole substitution. MG-II-20 has improved functional properties over previously examined variants, with Kd for gp120 in the nM range. In contrast, new variants of the Trp indole side chain, with either methyl- or bromo- components appended, had disruptive effects on gp120 binding, reflecting the sensitivity of function to changes in this component of the encounter complex. Plausible *in silico* models of cPT:gp120 complex structures were obtained that are consistent with the overall hypothesisof occupancy by the triazole Pro and Trp side chains, respectively, into the β20/21 and Phe43 sub-cavities. The overall results strengthen the definition of the cPT-Env inactivator binding site and provide a new lead composition (MG-II-20) as well as structure-function findings to guide future HIV-1 Env inactivator design.

## Introduction

HIV-1/AIDS remains a disease of globally epidemic proportions, with neither a protective vaccine nor a cure therapy available [1–3]. While available antiretroviral treatment therapies can suppress infection with improved duration of action, new treatments are needed to overcome the resistance to, and side effects of, current treatments and ultimately to enable a disease cure.

The HIV-1 surface Env protein complex is required for host cell infection by engaging both CD4 and co-receptor in tandem, and therein presents an enticing target for antagonist designs [4–6]. The peptide triazoles (PTs) we have identified are a unique class of such antagonist leads, which possess attractive antiviral functions, including the capacity to trigger Env gp120 shedding and consequently irreversible inactivation of virus [7–11]. Macrocyclic peptide triazoles (cPTs) have been devised with molecular weights of less than 1000 Da (comparable to small molecules), protease resistance, and greatly increased serum half-life [12, 13]. Furthermore, thiol functionalization of linear and cyclic PTs affords a subclass of variant PTs that cause further inactivation effects by virus membrane poration [14–17].

**Figure 1:**
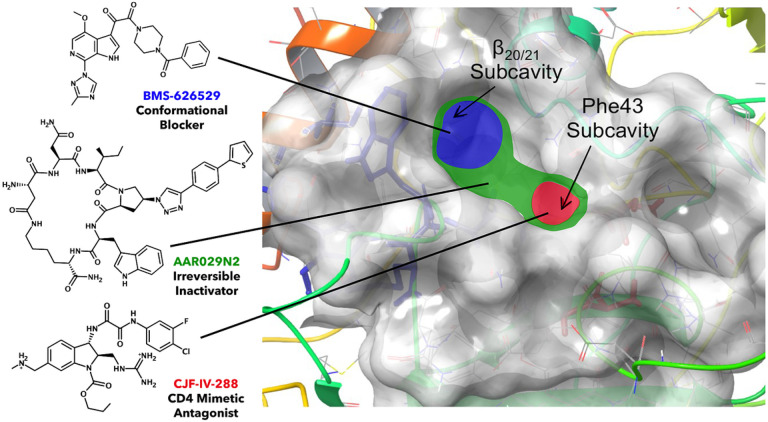
Differential utilization of two proximal subsites of the overall CD4 binding site in gp120. Space filling model of general subsite fitting of various HIV-1 entry inhibitors with three-color code of blue, red, and green. BMS-626529 (Temsavir, developed by Meanwell et al. [23]) and its main interaction site in the β20/21 subcavity are shown in blue. The CD4 mimetic CJF-IV-288 (developed by Fritschi et al. [24]) and its main interaction site in the Phe43 subcavity are shown in red. The macrocyclic peptide triazole AAR029N2 (developed by Rashad et al. [12, 13]) has been shown to interact with both subcavities, and both the cPT and its predicted interaction site are shown in green. The cPTs presented in this work are predicted to retain this dual-site interaction.

The structural underpinning of the antiviral functions of PT’s, including cPTs, is an Ile-TriazolePro-Trp pharmacophore (abbreviated as IXW) identified by a combination of synthetic variation, target gp120 sequence mutations, and genetic variations [18, 19]. Based on docking predictions and structure-function patterns, the triazolePro-R and Trp indole groups have been hypothesized to occupy two subsites of the overall CD4 binding site: one that is used by CD4 Phe43 and CD4 mimics, and a second that is used by temsavir-class inhibitors as potent conformational blockers (Fig. 1) [6, 20–22]. This dual subsite PT occupancy likely explains the dual CD4 and co-receptor site antagonism exhibited by the PTs, and the consequent unique antagonist functions of this class. However, the search for advancing synthetic designs has been impeded by the lack of high-resolution structure of cPT-gp120 complexes. As a result, important lingering questions regarding cPT pharmacophore structure-activity relationships have persisted. These include: (1) the organization of Triazole-Pro R substituent and Trp indole groups in the subsites of the gp120 CD4 binding interface; (2) whether the chemical space of these two PT groups can be further modified to improve antagonist potency; and (3) whether PT variants can be identified with improved potential as tools for high resolution structural definition of the cPT-gp120 interface.

In the current work, we addressed these SAR questions by a coordinated use of synthetic design, binding and antiviral analysis, and flexible docking. The data obtained have identified: (1) both subsite occupancy poses and stabilizing contacts for the key pharmacophore components; (2) a new lead MG-II-20; and (3) a Bromo-Trp variant as a potential crystallographic tool to localize the indole in cPT-gp120 complexes (using anomalous X-ray scattering), and thus to define better the structural nature of cPT-gp120 engagement.

## Results And Discussion

### Synthesis of Macrocyclic Compounds

We investigated new cPT designs in which the triazolePro R group and Trp indole side chains were varied in order to deepen our understanding of the role of these pharmacophore components in Env binding and antiviral functions. The variants of AAR029N2 were explored are shown in Fig. 2, which depicts a generalized synthetic scheme. The synthetic progression of compounds explored was guided by preliminary functional data from SPR and infection inhibition assays.

Details of the synthesis protocols are given in Materials and Methods, with validation chromatograms and mass spectra provided in **Figure S1A-F** (**Supplementary Material**). Sufficient amounts of all of the variants were obtained and used for binding and infection inhibition assays to define their functional properties.

### Kinetic and Affinity Binding Properties of cPT Inhibitor Variants

**Figure 3:**
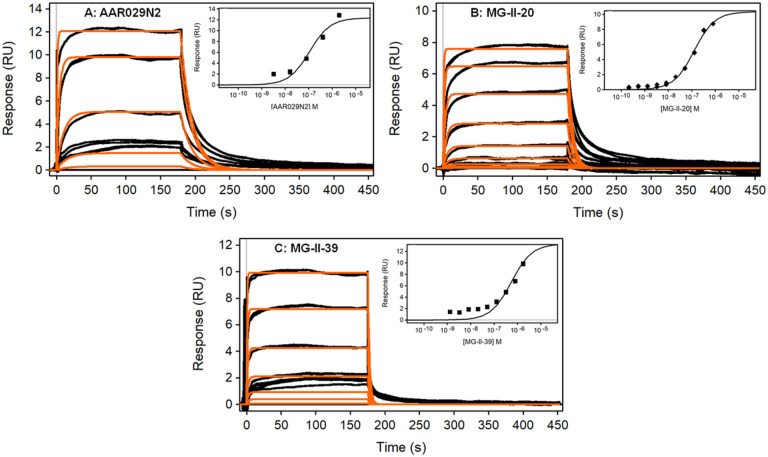
Sensorgrams for AAR029N2, MG-II-20 and MG-II-39 binding to YU2 gp120. Analyte samples in 5% dimethylsulfoxide (DMSO) running buffer were injected in duplicate assays at increasing concentrations, shown in (A) AAR029N2 (3.20 to 2000 nM, 5-fold dilutions); (B) MG-II-20 (8.2 to 800 nM, 2.5-fold dilutions); and (C) MG-II-39 (1.3 to 800nM, 2.5-fold dilutions), respectively (black traces). Overlay (red traces) corresponds to 1:1 model fit. Inset: steady state binding isotherm fits from 10^−10^ to 10^−5^ M reveal: A) K_D_= 110±2 nM; B) K_D_= 126.0±8 nM; C) K_D_= 517±30 nM. Ligand YU2 gp120: A) 3500RU; B) 1600 RU; C) 3500 RU.

A state-of-the-art Biacore S200 SPR optical biosensor, which can resolve dissociation rates of 0.1–1.0 s^−1^, was employed to measure the YU2 gp120 binding kinetics of cPT’s. Sensorgram data obtained for the parental N2 and two representative variant cases are given in Fig. 3, and for the remaining variants in **Figures S2-S4**. The insets in Fig. 3 show the binding isotherms plotted from the binding signals at steady state, as a function of peptide concentration. Reproducibility of the SPR binding assays was verified by running experiments at gp120 densities ranging from 10 to 29 RU/kDa. All kinetic and affinity data obtained from fitting binding sensorgrams to a 1:1 model are summarized in Table 1 below, and given in full detail in **Table S1**. The calculated apparent (KD), the steady state affinities (eq KD) and experimental Rmax or maximum binding capacities for each gp120 surface are reported.

Representative data in Fig. 3A show AAR029N2 binding to YU2 gp120, monitored for three minutes. The responses reached steady state within one minute and saturated the gp120 accessible sites between 800 and 2000nM concentrations. The dissociation phase was fast, within the first few seconds, and was complete after two minutes.

The kinetic results obtained show that MG-II-20 rates of gp120 binding are 1.5- to 2-fold higher, a significant change in the rate of complex formation compared to the parental AAR029N2. This is accompanied by an increase in the dissociation rate, which is significant in the presence of 2% dimethylsulfoxide (DMSO) (Table S1). The higher rate of association of MG-II-20 is evidenced in the saturation of the response relative to N2 in the same concentration range.

In contrast to the AAR029N2 and MG-II-20 cases, MG-II-39 (Fig. 3C) shows a lower affinity with displacement of the equilibrium binding isotherm toward higher concentrations (inset, 5-fold lower KD), and overall, faster association and dissociation rates than AAR029N2. In addition, the saturation of gp120 accessible sites is not attained in the 800–2000nM range for MG-II-39. Analyte concentrations were expanded around the KD obtained in the initial assays to determine as accurately as possible the affinities of MG-II-39, and the other cases for which very fast off-rates were observed.

The macrocyclic peptides MG-I-13 and MG-II-55, both containing a bromo-modified Trp indole, displayed very different kinetics with respect to each ‘parent’ peptide AAR029N2 and MG-II-20, respectively. **Supplemental Table S1** indicates that each bomo-Trp peptide binding to gp120 displayed a significant increase in association rate in 2% and 5% DMSO buffers, but also increases in the dissociation rates. A 2.5-fold increase in dissociation was measured for MG-I-13, while a 6-fold increase in dissociation rate was determined for MG-II-55 that significantly reduces the half-life of MG-II-55 bound complex from an average of 8.5 for MG-II-20 to 1.5 minutes (see **Supplemental Figures S2A and S2B**). The binding isotherm for MG-II-55 was derived from an analysis with an expanded number of analyte concentrations (as shown by the 1.3-fold analyte dilution series around the KD, **Figure S2B**). The kinetic distribution plot (**Figure S3**) depicts the relative changes in complex formation displayed by the cPT in 5% DMSO sample buffer. As shown by this “isoaffinity plot”, the binding kinetics for several of the cPT variants were found to be affected by changes in DMSO content in the SPR assays, though the relative affinities among the different variants generally were not affected.

### Inhibition of Pseudovirus-Cell Infection by Synthetic cPT Variants

**Figure 4:**
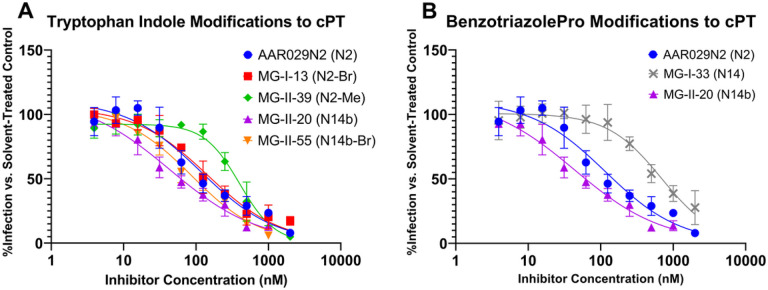
Dose responses of cPT variant infection inhibition against JRFL pseudotyped virus particles. (A) Infection inhibition profiles were measured for serial dilutions of AAR029N2 (N2), MG-I-13 (N2-Br), MG-II-39 (N2-Me), MG-II-20 (N14b), and MG-II-55 (N14b-Br). AAR029N2 IC_50_ = 116.4 ± 29.9 nM; MG-I-13 IC_50_ = 144.4 ± 27.0 nM; MG-II-39 IC_50_ = 399.9 ± 33.1 nM; MG-II-20 IC_50_ = 43.7 ± 16.4 nM; MG-II-55 IC_50_ = 87.1 ± 12.3 nM. (B) Infection inhibition profiles were measured for serial dilutions of AAR029N2, MG-I-33 (N14), and MG-II- 20 (N14b. Data and calculated IC_50_ values for AAR029N2 and MG-II-20 are reproduced from part A. MG-I-33 IC_50_ = 690.2 ± 86.4 nM. Data shown are the average of three independent experiments, and error bars indicate the standard deviation about the mean. Inhibition potencies are presented as the IC_50_ ± the standard error of the IC_50_, replicate wells = 4, independent experiments = 3.

Synthesized cPT compounds were evaluated for their inhibition potencies against JRFL pseudotyped virus particles in a cell-based infection assay. Serial dilutions of cPTs were incubated with JRFL pseudovirus for 30 minutes at 37°C, before addition to HOS.T4.R5 cells seeded the previous day at 10,000 cells/well in 96-well tissue culture plates. As indicated earlier, the selection of synthesized cPT compounds addressed two modifications in the IXW pharmacophore of the base cPT structure, exploring chemical space on the tryptophan (Fig. 4A) and varying the heterocycles affixed to the triazolePro R group (Fig. 4B).

Altering the triazolePro-coupled heterocycles resulted in varied changes to the infection inhibition potency. Replacing the thiophene of AAR029N2 with a difluoromethyl pyrazole (MG-I-33) resulted in a large loss of potency compared to AAR029N2. However, removal of the difluoromethyl, leaving a bare pyrazole in MG-II-20, restored and to some extent improved infection inhibition potency relative to AAR029N2.

Adding the bromine at the 5-position of the tryptophan indole resulted in small reductions in potency (AAR029N2 vs. MG-I-13 and MG-II-20 vs. MG-II-55 saw IC_50_ values increase by 30–40 nM). However replacing the indole nitrogen’s hydrogen with a methyl more significantly decreased inhibition potency (AAR029N2 vs. MG-II-39 increased in IC_50_ by ~ 280 nM).

### Computationally Derived Binding Models of cPTs Bound to HIV-1 Env gp120

**Figure 5:**
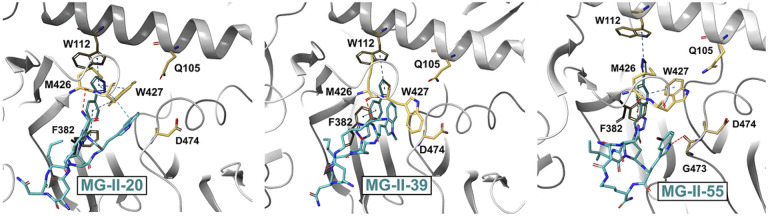
Putative binding mode of MG-II-20 (Left), MG-II-39 (Center), and MG-II-55 (Right) with gp120 obtained from the wild-type JRFL structure (PDB ID: 7N6U [28]). The triazole groups are uniformly located within the inner domain cavity and the tryptophan groups selectively interact in various modes with the Phe43 site. The cPT structures are shown as cyan sticks, H-bonds are shown as red dashed lines, and π-π stacking interactions are shown as blue dashed lines.

To assess further the cPT binding modes possible with dynamic HIV-1 Env gp120 and evaluate cPT pharmacophore engagement with the two subsite cavities (Fig. 1), we employed a Glide Induced Fit docking procedure to adjust active-site conformational flexibility *via* a protein structure prediction and refinement module (Prime [25–27]). The binding modes of MG-II-20, MG-II-39, and MG-II-55 obtained using as a target the gp120 protein extracted from the structure of a JRFL trimer, stabilized with BMS-378806 (PDB ID: 7N6U [28]) (Fig. 5), all predict that the triazolePro R moiety occupies the subsite cavity located under the β20/21 loop in similar orientations. Moreover, the bi-aromatic system of the triazolePro R moieties of both MG-II-20 and MG-II-55 makes three potential π-π stacking interactions with Phe382, Trp112, and Trp427 (Fig. 5**Left**, Fig. 5**Right**), while that of MG-II-39 makes two potential π-π stacking interactions with Phe382, and Trp112 (Fig. 5**Center**).

This occupancy reinforces an earlier prediction of the binding mode of the cPT benzothiophene triazole moiety of N2 with the gp120 subsite cavity located under the β20/21 loop [13]. Moreover, we noted that Trp427 makes three ring-ring interactions with the Trp indole moiety of MG-II-20, and one ring-ring interaction with the Trp indole moiety of MG-II-55. In contrast, the indole modification in MG-II-39, containing a methyl-indole moiety, shows no ring-ring interactions with Trp427. In the case of MG-II-39, the Trp Indole modification coincided with a reorientation of the Trp427 side-chain, while for MG-II-55, the Trp Indole moiety only interacts with G473 through hydrogen bonding. The changed subsite arrangements of both of these Trp variants coincided with loss of ring-ring interaction with Trp427. Generally, the different interactions of each of the cPT Trp Indole moieties with the Phe43 site reflect different utilization of the subsite cavity in proximity to α1-helix and α5-helix. This variability may reflect the dynamic state of this subsite cavity (Fig. 5). Alternatively, suboptimal engagement may occur with this cavity due to the bulkier tryptophan Indole moieties in MG-II-39 and MG-II-55. Nonetheless, the computational models are consistent with the intimate engagement of triazole-Pro R and Trp indole pharmacophore components respectively, with the β20/21 loop and Phe43 subsites of gp120.

### Insights on cPT Pharmacophore Usage in gp120 Encounter and Advancing SAR-Enhancing Synthetic Designs

The family of cPT variants investigated in this project incorporated a coordinated set of triazole Pro and Trp side chain modifications of the cPT IXW pharmacophore. The findings have enabled important insights into how the pharmacophore encounters the overall gp120 CD4 binding surface and provide important tools for deeper downstream structure-activity development and optimization.

### TriazolePro R Group Heterocycle Modifications

Significant refinement already has been accomplished for the triazolePro R group [9, 8, 11, 12, 20], with the thiophene of AAR029N2 providing the important N2 lead [13]. At the same time, the aryl R group opened up chemical space in this side chain for further synthetic design ideas. We explored the difluoromethyl pyrazole substitution of a thiophene (MG-I-33) early in the synthetic effort to determine fit of this bulkier group. Binding data (Table 1, **Figure S4**) showed that the MG-I-33 substitution decreased affinity substantially compared to AAR029N2. This was reflected further by a substantial loss of antiviral potency (Table 1, **IC**_**50**_), suggesting that the bulky and electronegative difluoromethyl group does not fit easily into the β20/21 cavity. This led to a trial with the unsubstituted pyrazole modification, with the latter retained based on a speculation that an aromatic nitrogen heterocyclic structure could potentially aid in binding, if bulk was reduced [8, 13, 29]. This speculation was confirmed, with MG-II-20 demonstrating promising improvements in both binding and antiviral assays. The improved activities may also signal the potential to improve targeting of the β20/21 subsite cavity. Computational modeling was consistent with a good fit of the triazolePro-benzopyrazole into the β20/21 subsite, so as to provide an associated fit of the pharmacophore Trp indole with the CD4 Phe43 subsite. From the perspective of structural fit, combined with enhanced infection inhibition potency vs AAR029N2, we believe MG-II-20 can be advanced as a productive lead.

### Tryptophan Indole Modifications

A key goal in the present study was to reinforce our understanding of the extent to which the Trp indole of the pharmacophore engages the CD4 binding interface of gp120. We found that modifications such as a methyl group or bromine group are disruptive of the binding process and have the corresponding major negative impact on antiviral function (see Table 1). Computational modeling suggests how this modification could alter Trp indole engagement (compare Fig. 5**Center** with 5 **Left** and 5 **Right**). In exploring two bromine-containing cPTs (MG-I-13 and MG-II-55), the impact of diminished activity (Table 1) and fit were both corroborated and clarified by molecular modeling (Fig. 5 and **Figure S5**). However, there are considerable differences between adding a bromine to the 5-position on the indole residue versus a methyl at the 1-position. Bromine addition to the Trp indole at the 5-position resulted in relatively minor decreases in binding and potency, possibly explained by potential steric clashes of bromo-tryptophan, but ameliorated by the dynamic state of the subsite cavity in the proximity of the α1-helix and α5-helix located at the interface between the outer and inner domains of gp120 unit. Methyl addition at the 1-position of tryptophan, on the other hand, resulted in larger binding and potency losses relative to the parent AAR029N2. This may have been due to impeding optimal utilization of the Phe43 subsite through the loss of a potential H-bond of the Trp indole nitrogen with the protein surface in MG-II-39, and disruption of π-π stacking with Env gp120’s Trp427. Notably, in the SPR analysis, all of the compounds bearing Trp indole modifications, whether bromine or methyl, featured faster on and off binding rates, possibly indicating a shallower and weaker binding interaction, and reinforcing the view that Trp427 plays a key role in cPT binding.

In addition to exploring the binding site occupancy of the pharmacophore Trp indole, bromo addition offers a potential advantage in crystallographic studies due to its detectability by anomalous X-ray scattering ([30] and Wayne Hendrickson, private communication). While bromo insertion in MG-I-13 was somewhat disruptive (Tables 1**and S1**), combining bromo insertion with the pyrazole substitution in the triazolePro R group led to the functionally improved MG-II-55.

## Conclusions

This work has yielded a new lead for cPT development, MG-II-20, through triazolePro R modification by substitution of a pyrazole for the previous thiophene. It has provided additional proof for the role of Trp indole in subsite occupancy by showing sensitivity to indole modifications, in particular methyl addition (MG-II-39). Computational modeling provided plausible structural rationales for the pyrazole’s strong fit in the β20/21 loop subsite, and for the Trp indole methyl and bromo additions’ disruptive impacts. Furthermore, the work allowed us to identify important tools for future structural studies (MG-II-20 and MG-II-55). Acquiring a high-resolution structure in future studies would significantly advance the structure-activity and design cycle effort to enhance potency and elevate the cPT class for downstream translational investigation.

## Materials And Methods

### Peptide synthesis

#### Materials

All reagents used in synthesis were obtained commercially from vendors such as CHEM-IMPEX, Alfa Aesar, Ambeed, and Sigma Aldrich and were high-performance liquid chromatography (HPLC) grade unless noted otherwise. The amino acid derivative of tryptophan with a bromine on the 5-position on the indole was obtained via the Smith lab at the University of Pennsylvania as described below. HPLC purifications were conducted with a Waters semi-preparative HPLC system and a Waters analytical HPLC system equipped with the Acquity QDa was used to perform purity checks and mass verification of synthesized peptides. All compounds were verified to greater than or equal to 95% homogeneity; yields are calculated based on purified product obtained.

#### Synthesis of Br-Trp

All reactions were conducted in oven-dried glassware under an inert atmosphere of nitrogen, unless otherwise stated. All solvents were reagent or HPLC grade. All reagents were purchased from commercially available sources and used as received. Reactions were magnetically stirred under a nitrogen atmosphere, unless otherwise noted and were monitored by thin layer chromatography (TLC) was performed on pre-coated silica gel 60 F-254 plates (40–55 micron, 230–400 mesh) and visualized by UV light or staining with KMnO_4_ and heating. Yields refer to chromatographically and spectroscopically pure compounds. Optical rotations were measured on a JASCO P-200 polarimeter. Proton (^1^H) and carbon (^13^C) NMR spectra (**Figure S6**) were recorded on a Bruker Avance III 500-MHz spectrometer. Chemical shifts (δ) are reported in parts per million (ppm) relative to methanol (δ 3.31) for ^1^H NMR, and methanol (δ 49.0). High resolution mass spectra (HRMS) were recorded at the University of Pennsylvania Mass Spectroscopy Service Center on either a VG Micromass 70/70H or VG ZAB-E spectrometer. The purity of new compounds was judged by NMR and LCMS (> 95%).

(−)-(S)-2-((((9H-fluoren-9-yl)methoxy)carbonyl)amino)-3-(5 bromo-1H-indol-3-yl)propanoic acid: 5-bromotryphophan (1) (4.0 g, 14.13 mmol, 1.0 equiv) was dissolved in dioxane (74 mL), and a solution of 10% w/v aq Na_2_CO_3_ (3.67 g, 2.45 equiv) was added. Following this, Fmoc-Cl (3.65 g, 14.13 mmol, 1.0 equiv) was dissolved in dioxane (45 mL) and added dropwise to the starting material solution at 0°C and allowed to warm up to room temperature overnight. Upon completion, the reaction was quenched by the addition of H_2_O (100 mL) and extracted with Et_2_O (100 mL). The aqueous layer was then acidified with concentrated HCl and extracted with EtOAc (3 × 100 mL). The organic layers were combined, washed with brine (100 mL), dried over Na_2_SO_4_, filtered, and concentrated in vacuo to yield crude (2). The crude compound was further purified via a crystallization with hot DCM to yield (2) as a white powder (6.8 g, 96% yield). ^1^H NMR (500 MHz, CD_3_OD) δ 7.79 (d, J = 8.2 Hz, 2H), 7.76 (d, J = 2.0 Hz, 1H), 7.59 (t, J = 6.6 Hz, 2H), 7.38 (t, J = 7.3 Hz, 2H), 7.27 (m, 3H), 7.19 (dd, J = 8.61, 1.82 Hz, 1H), 7.13 (s, 1H), 4.48 (q, J = 4.77, 3.47 Hz, 1H), 4.33 (dd, J = 6.7, 3.5 Hz, 1H), 4.24 (m, 1H), 4.19 (m, 1H), 1.09 (dd, J = 8.04, 6.3 Hz, 1H); ^13^C NMR (150 MHz, CD_3_OD) 175.46,158.44, 145.32, 145.25, 142.59, 142.55, 136.68, 130.78, 128.77, 128.75, 128.19, 126.39, 126.26, 126.07, 125.14, 121.95, 120.90, 120.86, 113.96, 113.10, 111.18, 68.09, 56.35, 48.40, 28.44; HRMS (ESI) m/z 505.0745 [calcd for C_26_H_21_BrN_2_O_4_ (M + H)^+^ 505.0763]; [α]_D_^24^−9.81 (c 1.05, MeOH).

#### Synthesis and purification of cPTs

All cPTs reported in this paper were synthesized via solid phase peptide synthesis using the Liberty Blue microwave peptide synthesizer (CEM, Matthews, NC) and other processes such as click conjugation. The synthesis scheme below follows the basic steps for obtaining the N2 cPT with necessary notes made for further derivatives. Firstly, a linear chain of six amino acids was made using the Liberty Blue at a 0.1M scale, with variations of the Trp-Br and Trp-Me for selected derivatives (MG-I-13, MG-II-39, MG-II-55). As stated previously, all amino acids were commercially obtained except for the Trp-Br. Then, using a 5% hydrazine solution in dimethylformamide (DMF), the Dde protecting group from lysine was removed via microwave irradiation on Liberty Blue to cyclize the peptide at the lysine and aspartic acid residues. After cyclization, an on-resin click-chemistry process of azide-alkyne cycloaddition was performed to obtain the triazole component of the cPT. The on-resin peptide was suspended in 8 mL DMF, 2 mL N,N-diisopropylethylamine, and 1 mL pyridine along with CuI (0.5 equiv.), L-ascorbic acid (0.6 equiv.), and 4-bromo-ethnylbenzene (5 equiv.). This step of the protocol occurred overnight in an external reaction vessel at room temperature, shaking for 18–20 hours. After the click-chemistry step occurred, the reaction vessel was drained and worked up with washes of 5% HCl and then DMF. The on-resin peptide was then suspended in DMF and transferred to the reaction vessel of the Liberty Blue with the desired Suzuki coupling reagent (1.2 equiv.), palladium catalyst (0.1 equiv.) and N,N-diisopropylethylamine (2 equiv.) for the Suzuki coupling step. Once completed, the on-resin cPT was deprotected using a cleavage cocktail of TFA, phenol, MilliQ water and TIPS (80%/5%/5%/10%). Once deprotected, the deprotection suspension was concentrated with a steady nitrogen gas stream, washed with cold diethyl ether, and then dried. The residue was dried and suspended in a 50/50 acetonitrile/H_2_O + 0.1% TFA buffer solution for purification.

All crude cPT solutions were purified via a preparative Waters HPLC system, using an XBridge CSH C18 column and a gradient of 90% H_2_O, 10% acetonitrile to 20% H_2_O and 80% acetonitrile, all HPLC solvents contained 0.1% TFA. The percentage yield ranged from 8%−12% depending on the ease of purification. Fractions collected were freeze dried using a lyophilizer and were resuspended in the 50/50 buffer to be verified for homogeneity and desired mass via analytical HPLC. This analytical HPLC system was by Waters as well and had a QDa to obtain mass chromatograms to validate the specific cPTs. All purified cPT chromatograms are provided in supplementary information, the UV/VIS analytical HPLC chromatograms were taken at 280nm via Waters 2489 UV/VIS detector. Prior to in vitro assays, all cPT samples were lyophilized and stored in a desiccator at room temperature. At times of assays, all compounds were dissolved in 100% DMSO at high concentrations to obtain stock solutions.

### Surface Plasmon Resonance Assays

#### Reagents

CM4 and CM5 carboxymethyldextran sensor chip series S (BR100012), sodium acetate solutions (pH 4, 4.5, 5.0 and 5.5, BR100349, BR100350, BR100351, and BR100352) and the amine coupling kit (BR100050) were purchased from (Cytiva Global Life Sciences Solutions, Marlborough, MA); sterile DMSO USP > 99.9% (Stemsol^™^, Protide Pharmaceuticals Inc., IL, USA) reagent grade (≥ 95%) phosphate salts, sodium chloride, tween-20 (or polysorbate-20 surfactant), hydrochloric acid, sodium hydroxide, and glycine, were purchased from Sigma-Aldrich (St. Louis, MO).

#### Expression and purification of wild-type YU-2 gp120 ligand

The DNA for gp120YU-2 in pcDNA3.1 vector for transient transfection was purified using a Qiagen MaxiPrep kit (Germantown, MD) and transfected into HEK-293F cells according to manufacturer’s protocol (Invitrogen). Five days after transfection was initiated, cells were harvested and spun down, and the supernatant was filtered through 0.2 μm filters. Purification was performed over a 17b antibody-coupled column prepared using an N-hydroxysuccinamide(NHS)-activated Sepharose, HiTrap HP column (GE Healthcare, Chicago, IL). Gp120 was eluted from the column using 0.1M Glycine buffer pH 2.4. The pH of the eluted protein was rapidly neutralized by addition of 1M Tris pH 8.0. Identity of the eluted fractions was confirmed by SDS-PAGE and Western blotting using antibody 16H3 (NIH HIV Reagent Program, Manassas, VA). Eluted protein was immediately buffer exchanged into PBS using spin-columns (Amicon Ultra Ultracell-30K, EMD Millipore, Burlington, MA). Protein was filtered through 0.45 μm syringe filters (Millex-LH, EMD Millipore) and separated by size exclusion on a HiLoad 26/60 Superdex 200 HR prepacked gel filtration column (GE Healthcare). Purity of eluted fractions and monomeric state of gp120 were identified by SDS-PAGE/Western blotting with mAb D7324. Monomeric fractions were pooled, concentrated, frozen and stored at −80°C.

#### Kinetic analysis of cPT binding to YU2 gp120

SPR experiments were performed on a Biacore S200 biosensor (Cytiva Global Life Sciences Solutions) at 25°C using PBS-P (10 mM Phosphate, 150 mM NaCl, pH 7.4, 0.005% P-20 and 2 or 5%DMSO) as the running and sample buffers. CM4 and CM5 sensor chips were docked and derivatized with gp120 YU2 after scouting the best coupling conditions varying preconcentration pH between 4.0 and 5.5; YU2 was coupled at 100μg/mL and pH 5.0 after activating each surface with freshly mixed 1:1 50 mM N-hydroxysuccinamide (NHS) and 200 mM 1-ethyl-3-(3-(dimethylamino) propyl) carbodiimide (EDC). Final YU2 densities were 10–25 RU/kDa (1200–3500RU). The 9 to 10 cPT concentrations were injected in duplicate spanning 1000-fold (~ 0.001 to 2.0 μM) at a flow rate of 75–100 μL/min where associations were monitored for 3 minutes and the final dissociation phase in running buffer monitored for 5 minutes for complete dissociation or by injecting two 9-second pulses of 10 mM glycine pH 2.0 to remove any bound analyte. The assay was repeated in at least two independent experiments for each cPT. The binding profiles were double referenced (subtraction of FC1 signal and the average of 6 duplicate buffer injections) to minimize the impact of instrument noise and baseline drift. Duplicate data sets were fit globally to a Langmuir 1:1 binding model using Scrubber 3.0c (BioLogic Software, Canberra, Australia) to calculate the kinetic constants *ka and kd*. The dissociation constant KD was calculated from the ratio of average kd/ka with the 95% confidence interval reported for n = 2. Steady state signal at each concentration was fit to a single binding isotherm to calculate the equilibrium KD.

### Infection Inhibition Assay

#### Pseudovirus Production

Recombinant HIV-1 pseudoviruses were produced from HEK-293T cells (CRL-3216, ATCC, Manassas, VA) as previously described [15, 12]. Cells were seeded in T75 flasks at 3 million cells per flask. Twenty-four hours after seeding, cells were transfected with 4 μg of HIV-1 JRFL envelope plasmid and 8 μg of backbone plasmid (pNL4 − 3.lucAM.R-E- developed by N. Landau and obtained from the NIH HIV Reagent Program, Manassas, VA). Forty-eight hours after transfection, virus-containing supernatants were collected, filtered through a 0.2 μm filter, and purified through a 6 − 20% iodixanol gradient, pooling fractions 6–9, out of 10. Virus aliquots were made, quick frozen on dry ice, then stored at −80°C.

#### Cell Infection Inhibition Assay

HOS.T4.R5 cells (ARP-3318, NIH HIV Reagent Program) were seeded in 96-well tissue culture plates at 10,000 cells per well, and cultured at 37°C with 5% CO_2_ for infection inhibition assays [31]. Aliquots of high-concentration cPT stocks in 100% DMSO were also diluted, stepwise, to 2% DMSO in DPBS (Corning, Corning, NY) warmed to 37°C in a water bath. Twenty-four hours after seeding, serial dilutions of cPT inhibitors were prepared in warmed 2% DMSO in DPBS and mixed in a 1:1 ratio with purified pseudovirus, and incubated for 30 min at 37°C, before addition to the plated cells. Twenty-four hours after addition of the cPT/virus mixture, growth medium exchange was performed on each plate. Twenty-four hours after medium exchange, HOS.T4.R5 cells were lysed using 1× lysis buffer (Promega) and 3 freeze-thaw cycles. Cell lysates were then transferred to white well plates (Greiner Bio-One), at 35 μL per well, and combined with 100 μL of luciferase buffer (15 mM MgSO_4_, 15 mM KPO_4_, 1 mM ATP, 1 mM DTT) and 50 μL of D-luciferin salt solution (1 mM solution in MilliQ water, Anaspec Inc., Fremont, CA), before measuring luminescence on a PerkinElmer 1450 Microbeta Liquid Scintillation and Luminescence Counter (Waltham, MA). Measured luminescence signals (in relative light units) were normalized to uninhibited pseudovirus treated with solvent only, and plotted as % infection. Data were plotted in GraphPad Prism 9, and IC_50_ values calculated using Prism’s nonlinear regression to a 4-parameter sigmoidal equation (GraphPad Software, Inc., San Diego, CA).

#### Molecular Modeling

The cryo-EM complex structure of wild-type JR-FL trimer in complex with BMS-378806 (PDB ID: 7N6U [28]) with resolution of 4.10 Å was downloaded from the RCSB protein data bank (https://www.rcsb.org/structure/7n6u). The initial structure was prepared with Schrödinger’s Protein Preparation Wizard [32–35, 27] via the following workflow tasks: adding hydrogens, assigning partial charges using the OPLS4 force field, and assigning protonation states. Following this step, the structure underwent energy minimization using the Impact [35] Refinement module and GPU-accelerated Desmond software [36–38], to let hydrogen atom positions freely relax while allowing for heavy atoms to relax strained bonds, angles, and clashes. The potential binding sites of extracted gp120 structure were explored and characterized using the SiteMap tool [39–41]. Then an identified binding site that includes the two subsite cavities was selected as the location of a docking grid box. The cPTs 3D molecular structures were preprocessed using LigPrep [42] to generate multiple states for stereoisomers, tautomers, and the protonation state. Then using the Macrocycle Conformational Sampling tool (Prime [25–27]) multiple conformers of cPTs were generated, and clustered using heavy atom RMSD. For each resulting 30 clusters, one conformer was selected as a cluster representative for the subsequent docking study. To consider the flexibility of both ligand and receptor in the docking study, the Induced Fit Docking (IFD) protocol [27, 43–47] was implemented. In IFD workflow, ligands were docked into the rigid protein using the soften-potential docking in Glide [47–50] with the van der Waals radii scaling of 0.8 for the proteins. The resulting top poses of each ligand were then used to sample the protein plasticity using the Prime [25–27]. Residues having at least one atom within 10 Å of any of the ligand poses were subject to a conformational search and energy minimization process to consider the flexibility of the binding site. The resulting new receptor conformations were taken forward for redocking. In this stage, Glide docking parameters were set to default hard-potential function. Glide SP (standard precision) was used for all the docking calculations. The returned poses with top Glide Score were then examined for putative binding modes.

## Figures and Tables

**Figure 2:** Synthetic cPT Variants and the Protocol for Their Synthesis. Bottom: the set of compounds investigated in this work focused on derivatives of AAR029N2 (shown on the left and highlighting the 3 pharmacophore residues, with modifications made in the Trp indole position (blue) and in the BenzoTriazole-Pro thiophene group (green). The isoleucine side chain (red), which is also part of the cPT pharmacophore, was not varied. Top: The general synthetic scheme used solid state peptide synthesis buildup, during which Trp or Trp variants were incorporated (blue), then cyclization followed by a Suzuki coupling step to incorporate triazole Pro R thiophene or thiophene replacements (green) using different commercially available boronic acids or boronic esters. The overall synthesis protocol was based on previous cPT syntheses [13].

**Figure 6:**
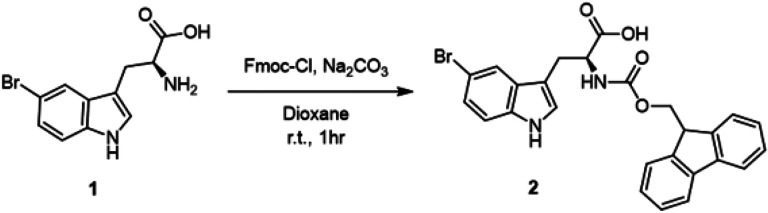
Unnumbered image in the Materials and Methods section.
